# Cracking the code: unveiling the nexus between atopic dermatitis and addictive behavior: a cross-sectional exploration of risk factors

**DOI:** 10.1007/s00403-024-02841-4

**Published:** 2024-03-14

**Authors:** Antonia Mick, Hannah Wecker, Stefanie Ziehfreund, Julia-Tatjana Maul, Tilo Biedermann, Alexander Zink

**Affiliations:** 1 Department of Dermatology and Allergy, Technical University of Munich, TUM School of Medicine and Health, Biedersteiner Str. 29, 80802 Munich, Germany; 2 Department of Dermatology, University Hospital of Zurich, Zurich, Switzerland; 3 Faculty of Medicine, University of Zürich, Zurich, Switzerland; 4 Division of Dermatology and Venereology, Department of Medicine Solna, Karolinska Institutet, Stockholm, Sweden

**Keywords:** Atopic dermatitis, Atopic eczema, Addictive behavior, Quality of life, Addiction

## Abstract

Atopic dermatitis (AD) stands as a prevalent chronic inflammatory skin disorder with a global reach. Beyond its cutaneous manifestations, AD is accompanied by comorbidities and psychological issues, significantly compromising the overall quality of life for individuals who suffer from AD. Previous research has evidenced a heightened prevalence of addictive disorders among dermatological patients when compared to the general population. Considering these findings, this study endeavors to examine the prevalence of addictive disorders among AD patients and, furthermore, to discern potential risk factors associated with this comorbidity. Therefore, a cross-sectional study was conducted involving patients with AD diagnosed by dermatologists within a large university hospital in Munich, South Germany, between January 2016 and December 2019. Patients received an anonymous paper-based questionnaire comprising standardized and reliable assessment tools concerning disease severity, quality of life, sexual dysfunction, well-being, and anxiety disorder as well as screening tools for various addictive disorders (compulsive internet use, drug abuse, pathological alcohol consumption, and smoking). Data were analyzed descriptively, and a multivariate logistic regression model was conducted. A total of 208 patients participated in the study, comprising 38% males and 62% females with a mean age of 44.8 ± standard deviation:17.9 years. Females showed a higher mean POEM (Patient-Oriented Eczema Measure) score compared to males (female 14.6 ± 7.8; male 12.5 ± 7.7), as well as a higher DLQI (Dermatology Life Quality Index) (female 8.5 ± 6; male 6.5 ± 6.5). Positive addictions were found in 14.9% for daily smoking, 15.4% for critical alcohol consumption, 16.8% for compulsive internet use, and 5.8% for drug abuse. Younger patients were more likely to be affected by one or multiple addictions than older patients. Patients with at least one addiction showed significantly impaired well-being and increased severe anxiety symptoms. Given the notable prevalence of addictive disorders among individuals with AD, it could be useful to implement systematic screening for such conditions as part of patient-centered care, especially focusing on young AD patients or those displaying concurrent indications of depression or anxiety.

## Introduction

Atopic dermatitis (AD) is one of the most common inflammatory skin diseases worldwide, as it affects about 20% of children and 1–3% of adults with an increase of incidence being observed [1, 2]. AD typically has its onset in early childhood, with ~ 70% of patients showing spontaneous remission before adulthood [2]. However, one in four patients reports AD onset during adulthood [3]. The pathogenesis of AD consists of a complex combination of genetic factors, environmental conditions, defective skin barrier function, and immunological factors, which makes the complete understanding of the disease challenging [4, 5]. AD is clinically characterized by chronic or relapsing eczema, dry skin, and pruritus [6]. AD is seen as the first step of the so-called atopic march, which can lead to other atopic comorbidities besides skin manifestations, such as asthma, allergic rhinitis, or food allergies [7, 8]. Additionally, studies have shown an association of AD with cardiovascular, malignant, and neuropsychiatric disorders; therefore, AD should be considered more of a systemic disease than a disease limited to the skin [9–12]. Due to its high incidence, systematic manifestations, chronic course, and therefore treatment, AD creates a high socioeconomic burden for individuals and society. A study has shown that each patient with moderate to severe AD incurs around €15,000 per year in direct and indirect costs due to loss of productivity [13]. Patients themselves also bear high annual out-of-pocket costs averaging 927€ in Europe [14]. Due to its high prevalence and the high social and financial costs, AD represents the largest burden of disability due to skin disease in the world [15].

The abovementioned multiple strains of AD have led to an increasing emphasis in research on the impact of this disease on the mental health of patients. AD has a major impact on the quality of life of patients, with greater disease activity being associated with lower happiness [16, 17] and poorer quality of life [18, 19]. Tsai et al. were able to show that there is a strong association between the occurrence of Attention Deficit Hyperactivity Disorder (ADHD) and AD in children [20]. AD patients also show an increased prevalence of depression, anxiety and suicidality [7, 21]. Furthermore, studies demonstrated that sexual dysfunction is commonly reported in patients with AD and represents another factor that reduces patients’ quality of life [22]. Although a direct causal association is lacking, AD’s chronic nature and impact on quality of life may potentially contribute to psychological distress [7]. This emotional burden may lead to coping mechanisms such as addictive behaviors, as already found in patients suffering from psoriasis, another chronic and life-impairing skin disease [23, 24]. Moreover, there is evidence of an increased prevalence of problematic alcohol consumption in patients with AD [25] and a German study showed first evidence of elevated positive screening rates for internet addiction and problematic gambling behavior in patients with AD compared to the general population [26].

However, no study has yet been conducted on possible risk factors that promote the occurrence of addictive disorders in patients with AD. Therefore, the aim of this study was to investigate the occurrence of addictions in patients with AD and to identify possible risk factors for the occurrence of addictive behaviors to screen and treat vulnerable groups in a patient-centered care setting for addictive behaviors.

## Material and methods

### Study design

In this non-interventional cross-sectional study, an anonymous survey of patients with AD was conducted at the Department of Dermatology and Allergy, Technical University of Munich in Germany. Paper-based questionnaires were sent to 1527 patients who presented at least once at the department of the Technical University of Munich between January 2016 and December 2019. The questionnaire was returned in a prepaid envelope. Inclusion criteria were an AD diagnosed according to International Classification of Diseases, tenth revision (ICD-10) and an age of 18 years or older. Furthermore, written informed consent was required to participate in the study and to allow processing of the data. The entire questionnaire is composed of several validated and standardized questionnaires. The paper questionnaires were digitized using REDCap (Research Electronic Data Capture, Vanderbilt University).

The study was reviewed and approved by the Ethics Committee of the Faculty of Medicine at the Technical University of Munich (248/20 S).

### Questionnaire

Patients were asked to indicate their age and sex. Additionally, weight and height were requested to calculate the body mass index (BMI). The patient-oriented eczema measure (POEM) was used to assess subjective disease severity. It considers the frequency of occurrence of seven symptoms (e.g., itching, sleep disturbance) over the last seven days, using a simple 5-point scale [27]. The score ranges from 0 to 28 points, with a higher score indicating greater severity. Dermatology Life Quality Index (DLQI) assesses patients' disease-related quality of life through ten questions [28]. Each question was rated from 0 to 3 points (not at all/not relevant, a little, a lot, very much), resulting in a maximum score of 30 points, with higher values indicating greater impairment of quality of life. The Drug Abuse Screening Test (DAST-10) is a ten-item questionnaire that is utilized for clinical screening of problematic substance use [29]. Responses scored with one point each for “yes” answers (except for item 3), contribute to a total score of 0–10 points, whereby a score of ≥ 3 is taken as cut-off value for a positive screening result [26, 30]. Patients who reported at least daily smoking, were classified as smokers. To detect alcohol abuse, the CAGE test (Cut down, Annoyed, Guilty, Eye-opener) was used. This questionnaire consists of four yes/no questions [31, 32]. If at least two questions were answered with "yes", alcohol consumption was classified as critical. The Short Compulsive Internet Use Scale (CIUS-5) consists of five questions which are answered on a five-point Likert scale from 0 "never" to 4 "very often". A score of ≥ 7 indicates problematic internet use [33]. The World Health Organization Well-Being Index (WHO-5) is a five-item measure for assessing well-being [34], which respondents rate on a 6-point scale from 0 (“at no time”) to 5 (“all of the time”). The  raw score thus ranges from 0 to 25. To obtain a percentage score ranging from 0 to 100, the raw score is multiplied by four with 0 representing the worst well-being and 100 the best [35].

The Generalized Anxiety Disorder Questionnaire (GAD-7), a seven-item questionnaire, assesses anxiety by referring to symptoms during the last two weeks (e.g., feeling nervous, trouble relaxing). Response options reached from "not at all” to "nearly every day" scored with 0 to 3 points, respectively [36]. Total scores vary from 0 to 21 points, with scores of 5, 10, and 15 representing cut-off points for mild, moderate, and severe anxiety [37]. The Arizona Sexual Experiences Scale (ASEX) was used to identify patients suffering from sexual dysfunction. The questionnaire consists of five questions about sexual experience (e.g., arousal, ability to reach orgasm), with each response giving 0 to 6 points. Scores range from 5 to 30 points, with a cut-off score of 19 or higher indicating sexual dysfunction (ASEX group). In addition, any one item with an individual score of ≥ 5 or any three items with scores of ≥ 4 also indicate sexual dysfunction [38, 39].

Initially, each addictive disorder was considered individually. Subsequently, two groups were formed: “any addiction” for those who screened positive for at least one addiction (e.g., tobacco addiction, alcohol addiction, computer addiction, drug abuse) and “no addiction” for all other patients.

Of the 1527 questionnaires which were sent out by mail 313 were returned. Of the 313 questionnaires returned, questionnaires with one missing item are excluded from analyses. Except from this were questionnaires with a maximum of one missing question in POEM and DLQI. These questionnaires are included, evaluating missing values as zero as indicated in the manual [40, 41]. Thus, a total of 208 patients have been included.

### *Statistical* analyses

The data were visually assessed for normal distribution using Q–Q plots, and descriptive statistics (mean, standard deviation (± sd), absolute numbers, proportions) were employed. Analyses were conducted for the entire study group and stratified by sex. To enhance the visualization of results, age groups were formed for the creation of figures instead of using metric age values. Group differences were examined using unpaired t-tests according to their normal distribution. If there were extreme outliers for a variable, the non-parametric Mann–Whitney-*U*-test was applied. The association of nominal variables was examined using the χ^2^ test. In addition, Pearson correlation (ρ) was applied for metric variables and Spearman correlation (*r*) for ordinal variables. A multivariate logistic regression model was calculated with the dichotomous outcome variable "any addiction" or "no addiction". Independent variables were age, sex (reference: women), BMI, POEM score, DLQI score, WHO-5 score, GAD-7 score, and ASEX group (reference: positive screened cases). The presence of multicollinearity was ruled out by correlation. Collinearity was assumed from a cut-off value of 0.8 in the correlation [42] and linearity of logit by examining the interaction of the variable and the logarithmized variable. If the results were not significant in the interaction, linearity of the logit can be assumed. Results of the multivariate logistic regression were reported with odds ratios (OR) and 95% confidence intervals (CI).

The level of significance was set at 0.05 and all statistical analyses were performed using the IBM SPSS Statistics Software for Mac (Version 28, IBM Corporation, Armonk, NY, USA).

## Results

### Patient characteristics

A total of 208 patients were analyzed, of whom 38% were male and 62% were female. Regarding all patients, the mean age was 44.8 ± 17.9 years, with female patients showing a younger mean age (40.4 ± 15.8) than male patients (52.1 ± 19). For the entire study population, a mean POEM of 13.8 ± 7.8 was observed, indicating a moderate eczema. Females showed a higher mean POEM score of 14.6 ± 7.8 than male patients with 12.5 ± 7.7 (*p* = 0.06). Female patients also had a significantly higher mean DLQI of 8.5 ± 6 points than males with a mean score of 6.5 ± 6 points (*p* = 0.03). Overall, the DLQI showed a moderate effect of the disease on the quality of life of the patients. The patients' well-being had a mean value of 53.2 ± 20.9 measured by WHO-5 score, with men (55.6 ± 21.7) showing slightly better well-being than women (51.7 ± 20.3, *p* = 0.19). A mean GAD-7 score of 6.6 ± 4.6 was observed, indicating mild anxiety, with males (5.8 ± 4.6) rated slightly less anxious than female patients (7.2 ± 4.6, *p* = 0.03, Table 1). Overall, 30.8% of patients were screened positive for sexual dysfunction. In comparison, 19% of men and 38% of women were affected (χ^2^(1) = 8.3, *p* = 0.004).

**Table 1:** Differences between sexes regarding various variables measured in patients with atopic dermatitis (N = 208)

Variables	Male (*n* = 79)	Female (*n* = 129)	Test statistics	*p*-value
Age, mean ± sd	52.1 ± 19	40.4 ± 15.8	*t*(206) = 4.82	< 0.001
BMI, mean ± sd	25.7 ± 4.2	23.9 ± 5.0	*z*(206) = − 3.65	< 0.001^a^
DLQI score, mean ± sd	6.5 ± 6.0	8.5 ± 6.0	*t*(206) = − 2.24	0.026
POEM score, mean ± sd	12.5 ± 7.7	14.6 ± 7.8	*t*(206) = − 1.89	0.060
ASEX group^b^, n (%)	15 (19%)	49 (38%)	χ^2^(1) = 8.3	0.004
WHO-5 score, mean ± sd	55.6 ± 21.7	51.7 ± 20.3	*t*(206) = 1.3	0.194
GAD-7 score, mean ± sd	5.8 ± 4.6	7.2 ± 4.6	*t*(206) = − 2.13	0.034

*sd* standard deviation, *BMI* Body Mass Index, *DLQI* Dermatology Life Quality Index, *POEM* Patient-Oriented Eczema Measure, *GAD-7* Generalized Anxiety Disorder Questionnaire, *WHO-5* WHO Well-Being Index, *ASEX* Arizona Sexual Experiences Scale

^a^Due to extreme outliers, Mann–Whitney-*U*-test was performed instead of *t*-test

^b^Cases of positive screened for sexual dysfunction, tested with χ^2^ test

### Addictions

In total, 39.4% were screened positive for at least one of the addictions assessed. Among these patients, 5.8% of patients were screened positive for critical drug use, 16.8% for compulsive internet use, 15.4% for critical alcohol consumption, and 14.9% were identified as daily smokers.

**Fig. 1:**
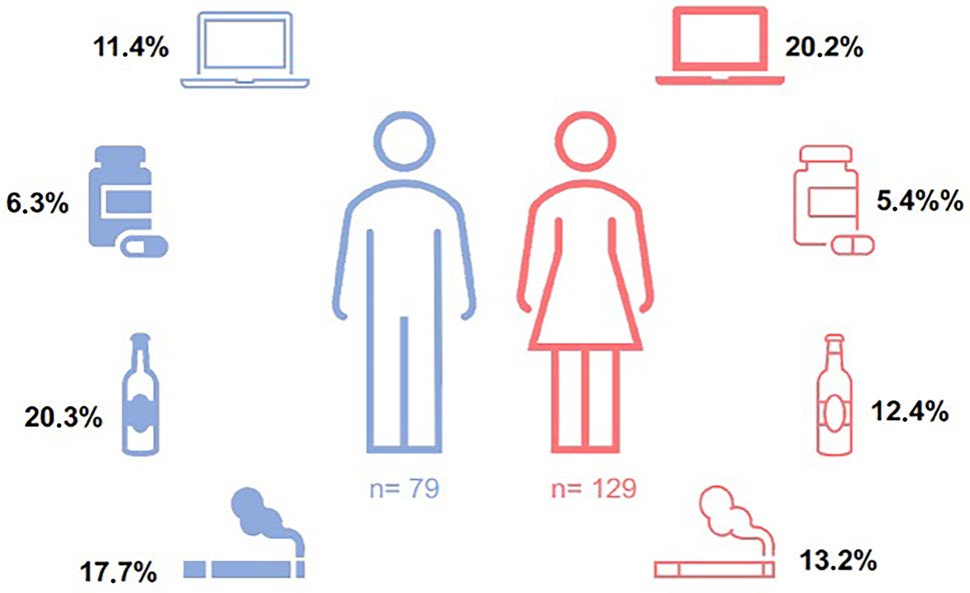
Occurrence of specified addictions (i.e. internet addiction, drug abuse, alcohol addiction, smoking) in patients with atopic dermatitis divided by sex

It was noticeable that females were more frequently screened positive for critical internet use (20.2% female; 11.4% male, χ^2^(1) = 2.7, *p* = 0.1). Men, by contrast, were more often positively screened for critical alcohol use (12.4% female; 20.3% male, χ^2^(1) = 2.3, *p* = 0.13, Fig. 1). In addition, patients exhibiting critical internet use were younger than patients without critical internet use (*t*(87) = 8.73, *p* < 0.001).

**Fig. 2:**
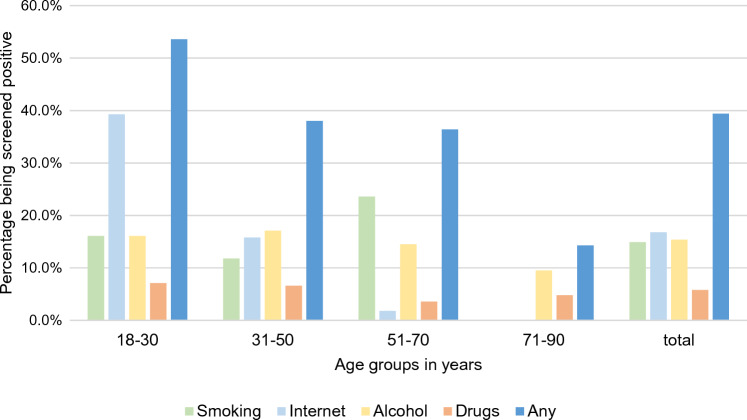
Occurrence of specified addictions and the occurrence of any addiction in patients with atopic dermatitis of different age groups and in total

More than one addiction was seen in 10.1% of patients. No patient was screened positive for all four addictions assessed. Almost no differences were found in the occurrence of multiple addictions between the sexes, with 11.4% among men and 9.3% among women (χ^2^(2) = 0.3, *p* = 0.86), whereas younger age was associated with the occurrence of multiple addictive disorders. (*r* = − 0.2, *p* = 0.004) (Fig. 2).

### Correlation between scores

Correlating the individual scores with each other, it was noticed that BMI correlated with GAD-7 score (ρ = − 0.15 *p* = 0.04). The DLQI was moderately to strongly correlated with POEM score (ρ = 0.64 *p* < 0.001), the WHO-5 score (ρ = − 0.43, *p* < 0.001), and GAD-7 score (ρ = 0.4, *p* < 0.001). Furthermore, POEM score showed a moderate correlation with WHO-5 score (ρ = − 0.34, *p* < 0.001) and GAD-7 score (ρ = 0.22, *p* = 0.001). WHO-5 score was strongly negatively correlated with GAD-7 score (ρ = − 0.65, *p* < 0.001). The number of addictive disorders present showed a weak to moderate correlation with WHO-5 score (ρ = − 0.12, *p* = 0.08) and GAD-7 score (ρ = 0.24, *p* < 0.001) in addition to correlation with age (ρ = − 0.21, *p* = 0.002).

### Any addiction vs. no addiction

The proportion of positively screened patients for "any addiction" showed with 39.2% for males and 39.5% for females almost no difference between sexes (χ^2^(1) = 0.1 *p* = 0.97, Table 2). Patients displaying at least one addiction were significantly younger than patients without addiction (any addiction 40.5 ± 16.7; no addiction 47.6 ± 18.2, *t*(206) = 2.85, *p* = 0.005). Moreover they showed higher disease severity as measured by POEM (“no addiction”: 13.6 ± 7.9, “any addiction”: 14.1 ± 7.7, *t*(206) = − 0.49, *p* = 0.62), more impaired quality of life according to DLQI (“no addiction”: 7.4 ± 6.0, “any addiction”: 8.3 ± 6.2, *t*(206) = − 1.06, *p* = 0.29) and a higher mean GAD-7 score (7.9 ± 4.7) compared to patients without addiction (5.8 ± 4.4, *t*(206) = − 3.23, *p* = 0.001). Additionally, patients with at least one addiction showed significantly lower WHO-5 scores (48.4 ± 21.2) compared to patients without any addiction (56.3 ± 20.1, *t*(206) = 2.72, *p* = 0.007). Among patients displaying addictive behavior, 23,6% were screened positive for sexual dysfunction, compared to 34,1% of patients without addiction (χ^2^(1) = 1.7, *p* = 0.19). Despite observed differences between these two groups, the logistic regression revealed significance only for age (OR = 0.98, 95% CI [0.96; 0.99], Table 3). However, the results from the group comparison were supported by the logistic regression: men, patients without sexual dysfunction, and increasing GAD-7 had a slightly higher risk of addiction, while patients with increasing BMI, disease severity, and well-being had a slightly lower risk of addiction.

**Table 2:** Differences between atopic dermatitis patients with at least one addiction and no addiction regarding various variables (*N* = 208)

Variables	No addiction (*n* = 126)	Any addiction (*n *= 82)	Test statistics	*p*-value
Age, mean ± sd	47.6 ± 18.2	40.5 ± 16.7	*t*(206) = 2.85	0.005
Sex (reference: women), *n* (%)	78 (61.9%)	51 (62.2%)	χ^2^(1) = 0.1	0.966
BMI, mean ± sd	24.7 ± 4.9	24.4 ± 4.5	*z*(206) = − 0.37	0.709^a^
DLQI Score, mean ± sd	7.4 ± 6.0	8.3 ± 6.2	*t*(206) = − 1.06	0.290
POEM Score, mean ± sd	13.6 ± 7.9	14.1 ± 7.7	*t*(206) = − 0.49	0.623
ASEX Group^b^, *n* (%)	43 (34.1%)	21 (25.6%)	χ^2^(1) = 1.7	0.193
WHO5 Score, mean ± sd	56.3 ± 20.1	48.4 ± 21.2	*t*(206) = 2.72	0.007
GAD-7 Score, mean ± sd	5.8 ± 4.4	7.9 ± 4.7	*t*(206) = − 3.23	0.001

*sd* standard deviation, *BMI* Body Mass Index, *DLQI* Dermatology Life Quality Index, *POEM* Patient-Oriented Eczema Measure, *GAD-7* Generalized Anxiety Disorder Questionnaire, *WHO5* WHO Well-Being Index, *ASEX* Arizona Sexual Experiences Scale

^a^Due to extreme outliers, Mann–Whitney-*U*-test was performed instead of *t*-test

^b^Cases of positive screened for sexual dysfunction, tested with χ^2^ test

**Table 3:** Results of multivariate logistic regression with the indicator of any addiction diagnosis as dependent dichotomous variable (“any addiction”, “no addiction”) and the independent variables age, sex, BMI, DLQI, POEM, WHO5, GAD-7, and ASEX group, presented as odds ratios and 95% confidence intervals (*N* = 208)

Variables	Odds ratio	95% confidence interval	*p*-value
Age	0.98	[0.96; 0.99]	0.020
Sex (reference: women)	1.38	[0.69; 2.75]	0.362
BMI	0.99	[0.93; 1.06]	0.871
DLQI Score	1	[0.94; 1.07]	0.996
POEM Score	0.99	[0.94; 1.04]	0.567
WHO-5 Score	0.99	[0.97; 1.01]	0.135
GAD-7 Score	1.07	[0.98; 1.16]	0.135
ASEX Group (reference: positive screened cases)	1.40	[0.65; 3.05]	0.392

*BMI* Body Mass Index, *DLQI* Dermatology Life Quality Index, *POEM* Patient-Oriented Eczema Measure, *GAD-7* Generalized Anxiety Disorder Questionnaire, *WHO-5* WHO Well-Being Index, *ASEX* Arizona Sexual Experiences Scale

## Discussion

In our cross-sectional study, a considerable screening rate of 39.4% for at least one addiction was observed in patients with AD. In addition, young patients in particular were found to be at increased risk, with internet addiction being particularly frequent. Furthermore, patients showing addictive behaviors had poorer well-being and were more likely to have symptoms of an anxiety disorder.

Sex-based differences in AD distribution and perception are well established. Regarding the distribution of AD, the general observation that more women are affected by it than men, especially in late-onset AD, is mirrored in the unequal distribution of sexes in our study population (62% female; 38% male) [43, 44]. In line with other studies, our study also showed that women experience stronger impairment of quality of life due to their disease than men, reflected in significantly higher mean DLQI and POEM scores [45]. Regarding further differences between the sexes, men exhibited better well-being than women as measured by WHO-5 scale and women showed a significantly higher GAD-7 score than men. As demonstrated before, AD patients exhibit an increased occurrence of depressive symptoms and anxiety compared to the general population [46, 47]. Furthermore, our study confirms significantly higher levels of depressive and anxiety symptoms in women with AD compared to men, consistent with existing literature on AD patients [48]. This sex discrepancy could potentially be attributed to the fact that visible skin lesions have a more pronounced impact on the quality of life of women than men [49], resulting in heightened psychological distress. Sexual health as an integral component of overall quality of is increasingly recognized in research on chronic skin diseases, including AD. Patients with chronic skin conditions, especially eczema, urticaria, or psoriasis, frequently experience impaired sexual life [50, 51]. Basson et al. (2018) have identified decreased mental health as the greatest risk factor for sexual dysfunction in women, possibly explaining the fact that in our study considerably more women were affected than men (19% men, 38% women) [52].

We identified 14.9% of patients as daily smokers, which is close to the rate of 15.1% in the healthy population in Germany [53]. In comparison, a significantly higher rate of 30.3% of daily smokers was shown for psoriasis, another chronic inflammatory skin disease [54].

Regarding critical alcohol use, 15.4% in our study had at least two positive responses on the CAGE questionnaire, indicating the likely presence of alcohol-related problems. In comparison, in another German study, only 8.1% of the healthy population reported having experienced two symptoms of the CAGE questionnaire [55]. A previously conducted study in AD patients showed critical alcohol use in 12.1% of patients [26]. Our study reinforces the previously established fact of more frequent critical alcohol use in AD patients, indicating even higher rates in our study. Individuals with chronic skin diseases often experience psychological distress, social anxiety, stigmatization, and low levels of self-esteem [25, 56]. The increased prevalence of alcohol abuse in AD patients could be explained according to the self-medication hypothesis to cope with psychological distress, aligning with the idea that individuals facing difficulties may resort to alcohol as a coping mechanism [57–59]. Our study found more critical alcohol behavior in males than females (12.4% female; 20.3% male), according to the general observation that women are less likely to show problematic drinking behavior [60].

A strikingly high percentage of 16.8% were screened positive for problematic internet use in our study, compared to considerably lower rates of 2.1% in the general German population and 4.5% in another study conducted on AD patients [26, 61]. A study on internet addiction in psoriasis patients reported increased screening rates of 8.5% compared to the general population, still lower than in our study on AD patients [24]. Despite the high rate of positively screened patients, more women than men were affected by internet addiction and those affected showed a significantly younger age. Other studies have shown either no difference between the sexes or that internet addiction is more likely to affect men than women, which we could not confirm with our results for patients with AD [62, 63]. A potential reason for the higher incidence of internet addiction among women in our study could be their younger average age compared to male participants, as the increased incidence of compulsive internet use among younger people was already shown in other studies [64]. It must be mentioned that the definition of internet addiction is not uniform and there are different assessment tools, which can make comparability difficult. Prior research demonstrates a strong link between mental health and quality of life with the occurrence of internet addiction [65]. The reduced quality of life and mental health in AD patients could, therefore, potentially explain the elevated screening rates for internet addiction in our study.

For substance abuse, 5.8% of the study population screened, comparable to the percentage of 6% shown by Schielein et al. for patients suffering from psoriasis [54]. In contrast, a value of only 3% for substance-related disorders was found in the general population of Germany [53].

When comparing the two groups, we could see that patients in the group "any addiction" showed a lower mean age, worse well-being, and higher levels of anxiety. The clinical assessment of disease severity does not always correlate with the patient's perception of illness in dermatologic diseases. It is possible that psychological and social determinants could exert a greater influence on quality of life [66, 67]. Although mean scores for POEM and DLQI were slightly higher in patients with positive addictive behavior, these differences were not significant in our study. Another study conducted on addictive behavior in patients with AD also did not find a correlation between POEM and addictive behavior in general but identified a significant link between DLQI score and the number of cigarettes smoked [26]. Correspondingly, a study on addictive behavior in psoriasis patients showed that PASI, a severity score for psoriasis, was only significantly associated with smoking [54]. This observation supports that the severity of AD may not be the primary determinant of addictive behavior development. Instead, the risk appears to be rather influenced by a younger age or the presence of anxiety or depression as comorbidities.

## Limitations

In terms of limitations, our study population may not be fully representative of German AD patients, as only patients who have been treated at a university hospital were included. These patients may therefore show a higher disease severity. Furthermore, our study population showed an uneven age distribution between the sexes. Additionally, our study lacks an objective measure of disease severity and psychological symptoms, relying solely on self-assessment for data collection. Additionally, it should be noted that a uniform definition for sexual dysfunction is lacking, and this study adopts the definition based on the ASEX questionnaire. Moreover, our study involved the collection of highly sensitive and personal data. There is a potential for social desirability bias, where a sense of shame or the desire for social approval may have influenced certain patients to withhold completely truthful responses to the questions [68]. Furthermore, we do not have a control group, so that only indirect comparison with other studies was possible. Finally, it should be mentioned that no onset or time frame for AD, addiction, or other comorbidities was documented, so the direct or indirect nature of the association between these conditions cannot be addressed.

## Conclusion

This comprehensive study provides evidence of a higher prevalence of addictive disorders, excluding smoking, in AD patients compared to the general population. Particularly, internet addiction appears to be prevalent in the younger age group, raising concerns about this specific population. Interestingly, the findings suggest that mental health comorbidities, notably anxiety and depression, exert a greater influence on the development of addictive behavior than the severity of the individual's skin disease. Consequently, this high-risk group of AD patients should be subject to screening for addictive disorders, as part of optimizing patient-centered care. Screening becomes particularly pertinent for younger AD patients and those presenting with symptoms of depression or anxiety, warranting additional investigation in larger-scale clinical studies to further elucidate the dynamics at play.

## Data Availability

The data that support the findings of this study are available from the corresponding author, Alexander Zink, upon reasonable request.
